# Risk of systemic rheumatic diseases in people with irritable bowel syndrome: a global-federated cohort analysis

**DOI:** 10.3389/fimmu.2026.1779191

**Published:** 2026-08-03

**Authors:** Shuo-Yan Gau, Yu-Jung Su, Shih-Chi Yang, Chien-Chin Chen, Solomon Chih-Cheng Chen, Hui-Chin Chang, Meng-Che Wu

**Affiliations:** 1 Department of Medical Education, Ditmanson Medical Foundation Chia-Yi Christian Hospital, Chiayi, Taiwan; 2 Institute of Allergology, Charité-Universitätsmedizin Berlin, Corporate Member of Freie Universität Berlin and Humboldt-Universität zu Berlin, Berlin, Germany; 3 Department and Graduate Institute of Business Administration, National Taiwan University, Taipei, Taiwan; 4 Orthopedics Department, Chi-Mei Medical Center, Tainan, Taiwan; 5 Education Center, National Cheng Kung University Hospital, College of Medicine, National Cheng Kung University, Tainan, Taiwan; 6 Department of Pathology, Ditmanson Medical Foundation Chia-Yi Christian Hospital, Chiayi, Taiwan; 7 Department of Cosmetic Science, Chia Nan University of Pharmacy and Science, Tainan, Taiwan; 8 Ph.D. Program in Translational Medicine, Rong Hsing Research Center for Translational Medicine, National Chung Hsing University, Taichung, Taiwan; 9 Department of Biotechnology and Bioindustry Sciences, College of Bioscience and Biotechnology, National Cheng Kung University, Tainan, Taiwan; 10 Department of Pediatrics, Ditmanson Medical Foundation Chia-Yi Christian Hospital, Chiayi, Taiwan; 11 Department of Pediatrics, College of Medicine, Taipei Medical University, Taipei, Taiwan; 12 Evidence-based Medicine Center, Chung Shan Medical University Hospital, Taichung, Taiwan; 13 Library, Chung Shan Medical University Hospital, Taichung, Taiwan; 14 School of Medicine, Chung Shan Medical University, Taichung, Taiwan; 15 Division of Pediatric Gastroenterology, Children’s Medical Center, Taichung Veterans General Hospital, Taichung, Taiwan; 16 Department of Post-Baccalaureate Medicine, College of Medicine, National Chung Hsing University, Taichung, Taiwan

**Keywords:** electronic health records, immunology, irritable bowel syndrome, rheumatic diseases, TriNetX

## Abstract

**Background:**

Irritable bowel syndrome (IBS) is a prevalent disorder of gut–brain interaction increasingly linked to immune and inflammatory dysregulation. Whether IBS predisposes to systemic rheumatic diseases remains uncertain.

**Methods:**

We conducted a retrospective cohort study using the TriNetX Global Collaborative Network, which integrates anonymized electronic health records from more than 150 healthcare organizations and 150 million patients worldwide. Adults (≥18 years) diagnosed with IBS (≥2 visits) between 2015 and 2023 were compared with matched controls undergoing routine health evaluations without IBS. Exclusion criteria included malignancy, pre-existing systemic rheumatic diseases, and death before the index date. Propensity score matching (1:1) balanced demographics, comorbidities, psychiatric disorders, and socioeconomic factors. The primary outcome was incident systemic rheumatic disease, categorized into inflammatory arthritis (ankylosing spondylitis, psoriatic arthritis, rheumatoid arthritis, gout) and connective tissue disorders (systemic lupus erythematosus, systemic sclerosis, Sjögren syndrome, dermatomyositis/polymyositis). Risk estimates were generated using the TriNetX analytic function, reporting hazard ratios with 95% confidence intervals. Sensitivity analyses included alternative definitions, extended washout periods (24 and 36 months), varying follow-up durations, active comparator cohorts, and negative controls. Cross-dataset validation was performed using the TriNetX US Collaborative Network.

**Results:**

After matching, 459966 patients were included in each cohort. Irritable bowel syndrome was associated with increased risks of ankylosing spondylitis (HR 2.57, 95% CI 2.22–2.98), psoriatic arthritis (HR 2.03, 95% CI 1.80–2.29), rheumatoid arthritis (HR 1.42, 95% CI 1.28–1.56), and gout (HR 1.34, 95% CI 1.27–1.42). Elevated risks were also observed for systemic lupus erythematosus (HR 1.84, 95% CI 1.65–2.04), Sjögren syndrome (HR 2.84, 95% CI 2.60–3.12), systemic sclerosis (HR 2.09, 95% CI 1.64–2.67), and dermatomyositis (HR 1.52, 95% CI 1.11–2.07). These associations remained across multiple sensitivity and stratified analyses.

**Conclusions:**

IBS was associated with a higher risk of diverse systemic rheumatic diseases. Further studies are warranted to clarify the mechanisms underlying these observed associations.

## Introduction

Irritable bowel syndrome (IBS) is a common disorder of gut–brain interaction characterized by recurrent abdominal pain associated with altered bowel habits in the absence of structural disease. It is diagnosed according to symptom-based criteria, such as the Rome framework (1). Population-based surveys indicate that IBS affects approximately 10% of the global population, with a substantial female predominance and considerable regional variability (2). Beyond gastrointestinal symptoms, IBS is associated with impaired health-related quality of life and substantial indirect costs related to work absenteeism and presenteeism (3, 4).

Accumulating evidence suggests that IBS frequently coexists with immune-mediated and rheumatic conditions (5–7). Population-based studies have reported a higher risk of IBS among individuals with atopy, autoimmune diseases, and other chronic immune disorders than among community controls (8, 9). In patients with systemic lupus erythematosus (SLE), IBS-like symptoms are common and are associated with poorer quality of life and greater fatigue, suggesting overlapping pathophysiological mechanisms involving functional bowel symptoms and immune-mediated inflammatory pathways (10). Furthermore, recent evidence has documented an increased burden of IBS among patients with spondyloarthritis, underscoring clinically relevant gut–joint interactions (11).

Despite these observations, the temporal association between IBS and subsequent systemic rheumatic diseases remains incompletely characterized. Most prior studies have evaluated IBS-type symptoms in patients with established rheumatic diseases or have focused on selected conditions such as rheumatoid arthritis (RA), SLE, or spondyloarthritis (8, 11). Recent evidence has also highlighted that rheumatologic comorbidities are more commonly observed in patients with inflammatory conditions such as inflammatory bowel disease (IBD), suggesting a potential link between chronic inflammation and systemic rheumatic manifestations (12); however, whether a similar association exists for IBS, which lacks overt inflammatory pathology, remains unclear. Less is known about whether individuals diagnosed with IBS subsequently experience a higher risk of a broader range of systemic rheumatic diseases in routine clinical practice. This question is clinically relevant because symptoms attributed to IBS may coexist with nonspecific systemic manifestations, while delayed recognition of rheumatic diseases may adversely affect long-term outcomes.

Therefore, we conducted a large-scale retrospective cohort study using the TriNetX Global Collaborative Network. The primary objective was to examine the association between IBS and incident systemic rheumatic diseases, covering both inflammatory arthritis and connective tissue disease categories. We further evaluated the consistency of these associations through alternative IBS definitions, extended washout periods, active comparator cohorts, negative-control outcomes, subgroup analyses, and cross-dataset validation.

## Materials and methods

### Study design and data source

We conducted a retrospective cohort study using real-world data from the TriNetX Global Collaborative Network. This international research platform aggregates continually updated, anonymized electronic health records (EHRs) from more than 150 participating healthcare organizations across multiple continents, representing a patient population of over 150 million individuals. TriNetX has been widely used in previous epidemiological, health economics, and outcomes research (13–15).

### Cohort definition

The study cohorts were defined using specific diagnostic, procedural, and medication codes (detailed in **Supplementary Table 1**) recorded between 2015 and 2023. The IBS cohort comprised patients with a documented diagnosis of IBS who had attended at least two healthcare visits. The control cohort consisted of adults undergoing routine health evaluations with no documented diagnosis of IBS. Exclusion criteria included age younger than 18 years, any diagnosis of inflammatory bowel disease, a history of malignancy, pre-existing systemic rheumatic disease, or death before the index date. Individuals with inflammatory bowel disease were excluded regardless of the timing of diagnosis to minimize potential disease overlap and diagnostic ambiguity.

### Outcomes

The primary outcomes were incident systemic rheumatic diseases recorded after the index date. These outcomes were grouped into inflammatory arthritis and connective tissue disease categories. Inflammatory arthritis outcomes included ankylosing spondylitis, RA, psoriatic arthritis, and gout. Connective tissue disease outcomes included SLE, Sjögren syndrome, systemic sclerosis, and dermatomyositis/polymyositis. The International Classification of Diseases, 10th Revision, Clinical Modification (ICD-10-CM) codes used for exposure, outcomes, covariates, and sensitivity analyses are provided in **Supplementary Table 1**.

### Covariates and propensity score matching

To improve baseline comparability, IBS patients and controls underwent 1:1 propensity score matching. Matching was performed using greedy nearest-neighbor matching without replacement and a caliper of 0.10 pooled standard deviations. The propensity score model included age, sex, race, body mass index, hypertension, hyperlipidemia, diabetes mellitus, psychiatric conditions coded within ICD-10-CM F40–F48, and mental or behavioral disorders related to psychoactive substance use coded within F10–F19. Medication exposure was reported descriptively in the baseline table but was not included in the primary propensity score model. Covariate balance was assessed using standardized mean differences, with an absolute value below 0.1 considered acceptable. Body mass index was operationalized as a binary variable indicating a recorded value of at least 25 kg/m². Missing BMI values were not imputed. Patients with unavailable race information were retained within the platform-defined “unknown race” category.

### Sensitivity and comparator analyses

Several sensitivity analyses were performed to examine the consistency of the findings (**Supplementary Table 2**). Alternative IBS definitions were applied, including repeated IBS diagnosis within 30 days, more than five IBS diagnostic records, IBS with abdominal pain, and IBS with change in bowel habit. Additional models used shorter follow-up windows and extended washout periods of 24 and 36 months. To address potential diagnostic ambiguity from other gastrointestinal disorders, additional analyses excluded individuals with a history of cholecystectomy or selected abdominal disorders that may mimic IBS. These disorders included celiac disease, microscopic colitis, diseases of the esophagus, stomach, and duodenum, and diseases of the peritoneum and retroperitoneum. Active comparator analyses were performed using patients with hernia and patients with gastritis or duodenitis as alternative control groups. The gastritis or duodenitis comparator was used as a gastrointestinal healthcare-seeking comparison group rather than as a true negative-control exposure. Negative-control outcome analyses were conducted using tuberculosis and rickettsioses. To further address differential healthcare utilization, an additional propensity score model included platform-defined visit variables. These variables consisted of any prior ambulatory visit and any prior inpatient encounter recorded in TriNetX. They were assessed using all available pre-index records through the day before the index date and were not based on specific diagnostic or procedural codes. The TriNetX interface did not provide visit counts, specialty-specific encounter information, referral patterns, or laboratory testing intensity.

### Subgroup analyses

Subgroup analyses were performed according to age, sex, and race. Propensity score matching was repeated separately within each sensitivity and stratified analysis.

### Statistical analysis

All statistical analyses were performed within the “compare outcomes” module of the TriNetX analytic platform. Information regarding data access and analysis date were presented in the Supplementary statement. Time-to-event outcomes were evaluated using the built-in TriNetX survival analysis module, which generated hazard ratios (HR) with 95% confidence intervals (95% CI), Kaplan–Meier estimates, and log-rank p values. Risk ratios and risk differences were also reported for the primary analysis to provide absolute risk context. The underlying model specification was not accessible to investigators, and formal testing of the proportional hazards assumption was unavailable in the present analysis. Individual-level censoring dates and details of the censoring algorithm were likewise unavailable through the federated interface. No formal *a priori* power calculation was performed because this retrospective database study included all eligible patients identified through the TriNetX Global Collaborative Network according to prespecified criteria. The TriNetX federated interface did not provide a dedicated power-analysis function or individual-level data access required for an independent formal time-to-event power calculation. Statistical precision was therefore assessed using observed event counts and 95% confidence intervals, and absolute risk measures were reported to aid interpretation. Death recorded before the index date was treated as an exclusion criterion. Death occurring during follow-up was not incorporated as a competing event, and no competing-risk analysis was performed. Continuous variables were reported as mean ± standard deviation when available from the TriNetX output. Categorical variables were presented as number followed by percentage. Baseline covariate balance after propensity score matching was assessed using standardized mean differences rather than parametric or nonparametric hypothesis tests, with an absolute value below 0.1 considered acceptable.

### Ethical approval

Access to the Global Collaborative Network was utilized for this research. The study adhered to the ethical stipulations outlined in the Declaration of Helsinki and was granted a waiver of informed consent by the Institutional Review Board of Ditmanson Medical Foundation Chia-Yi Christian Hospital (IRB No. IRB2025121).

## Results

### Study population and baseline characteristics

**Figure 1:**
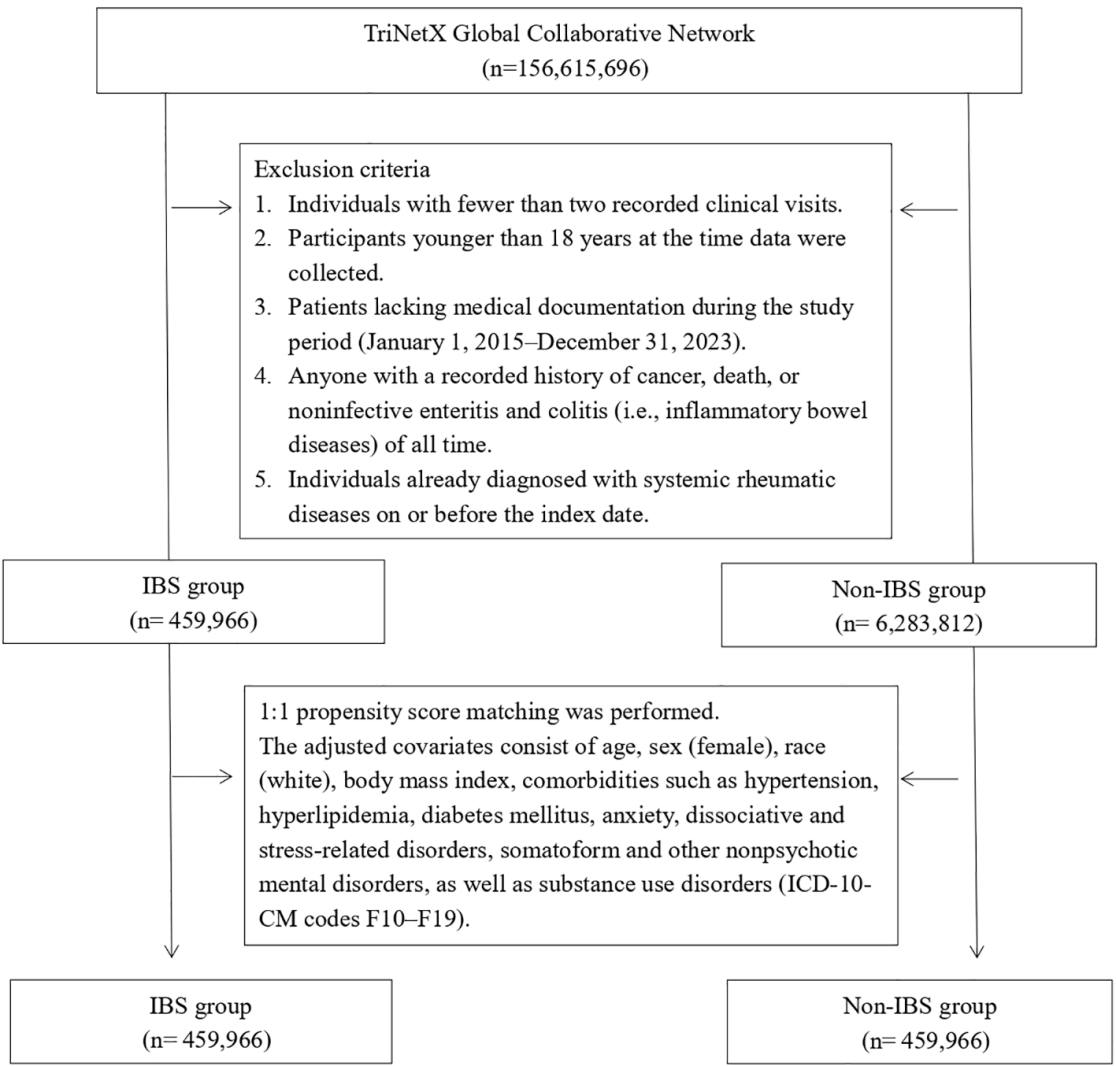
Flowchart of patient selection and cohort construction. Flowchart showing the identification of eligible adults from the TriNetX Global Collaborative Network between 2015 and 2023. Patients with irritable bowel syndrome (IBS) and individuals without a recorded IBS diagnosis were screened according to predefined inclusion and exclusion criteria. Individuals younger than 18 years or with inflammatory bowel disease, malignancy, pre-existing systemic rheumatic disease, or death before the index date were excluded. After 1:1 propensity score matching, 459,966 patients were retained in each cohort for the primary analysis.

From 156,615,696 adults in the TriNetX Global Collaborative Network, 459,966 individuals with IBS and 6,283,812 individuals without IBS met the eligibility criteria. After 1:1 propensity score matching, 459,966 patients were retained in each cohort for the primary analysis (**Figure 1**).

Before matching, patients with IBS were more often female and White, and they had higher frequencies of anxiety-related disorders and selective serotonin reuptake inhibitor use. After matching, most baseline characteristics were well balanced between cohorts, with only small residual differences in selected race categories and healthcare utilization variables (**Table 1**).

**Table 1:** Baseline demographic and clinical characteristics of patients with and without irritable bowel syndrome before and after 1:1 propensity score matching.

	Before matching			After matching		
	IBS cohort (n=459,966)	Control cohort (n=6,283,812)	SMD	IBS cohort (n=459,966)	Control cohort (n=459,966)	SMD
Age at index						
Mean±SD	44.7±18.9	43.2±18.3	0.08	44.7±18.9	44.7±18.9	0.00
Sex						
Male	127727 (27.8)	2764418 (44.0)	0.34	127727 (27.8)	126855 (27.6)	0.00
Female	331852 (72.1)	3496674 (55.6)	0.35	331852 (72.1)	331853 (72.1)	0.00
Unknown Gender	387 (0.1)	22720 (0.4)	0.06	387 (0.1)	1258 (0.3)	0.04
Race, n (%)						
White	293269 (63.8)	3795904 (60.4)	0.07	293269 (63.8)	293309 (63.8)	0.00
Black or African American	29944 (6.5)	850969 (13.5)	0.24	29944 (6.5)	51385 (11.2)	0.16
Asian	19805 (4.3)	271824 (4.3)	0.00	19805 (4.3)	18313 (4.0)	0.02
Native Hawaiian or other Pacific Islander	626 (0.1)	16947 (0.3)	0.03	626 (0.1)	1108 (0.2)	0.02
American Indian or Alaska Native	1272 (0.3)	21365 (0.3)	0.01	1272 (0.3)	1452 (0.3)	0.01
Unknown Race	93845 (20.4)	976657 (15.5)	0.13	93845 (20.4)	70585 (15.3)	0.13
Other Race	21205 (4.6)	350146 (5.6)	0.04	21205 (4.6)	23814 (5.2)	0.03
Medical Utilization Status						
Ambulatory visit	366822 (79.8)	5107288 (81.3)	0.04	366822 (79.8)	375750 (81.7)	0.05
Emergency visit	119780 (26.0)	1531045 (24.4)	0.04	119780 (26.0)	111821 (24.3)	0.04
Inpatient encounter	89851 (19.5)	960921 (15.3)	0.11	89851 (19.5)	74061 (16.1)	0.09
Lifestyle						
Mental and behavioral disorders due to psychoactive substance use	36447 (7.9)	475752 (7.6)	0.01	36447 (7.9)	36436 (7.9)	0.00
Socioeconomic status						
Persons with potential health hazards related to socioeconomic and psychosocial circumstances	7361 (1.6)	112329 (1.8)	0.01	7361 (1.6)	9838 (2.1)	0.04
Family history						
Family history of arthritis and other diseases of the musculoskeletal system and connective tissue	1252 (0.3)	10875 (0.2)	0.02	1252 (0.3)	1030 (0.2)	0.01
BMI, n (%)						
≧ 25 (kg/m2)	142526 (31.0)	2133398 (34.0)	0.06	142526 (31.0)	142533 (31.0)	0.00
C reactive protein, n (%)						
≧ 5 (mg/L)	20414 (4.4)	143474 (2.3)	0.12	20414 (4.4)	11082 (2.4)	0.11
Comorbidities						
Essential hypertension	76862 (16.7)	1095075 (17.4)	0.02	76862 (16.7)	76873 (16.7)	0.00
Hyperlipidemia	44435 (9.7)	645883 (10.3)	0.02	44435 (9.7)	44417 (9.7)	0.00
Diabetes mellitus	29361 (6.4)	405196 (6.4)	0.00	29361 (6.4)	29334 (6.4)	0.00
Anxiety, dissociative, stress-related, somatoform and other nonpsychotic mental disorders	101078 (22.0)	800185 (12.7)	0.25	101078 (22.0)	101062 (22.0)	0.00
Major depressive disorder, recurrent	16007 (3.5)	130005 (2.1)	0.09	16007 (3.5)	13823 (3.0)	0.03
Vitamin D deficiency	32041 (7.0)	372023 (5.9)	0.04	32041 (7.0)	31397 (6.8)	0.01
Sleep apnea	20222 (4.4)	201687 (3.2)	0.06	20222 (4.4)	13649 (3.0)	0.08
Chronic ischemic heart disease	14020 (3.0)	169054 (2.7)	0.02	14020 (3.0)	10712 (2.3)	0.04
Chronic kidney disease	7837 (1.7)	98278 (1.6)	0.01	7837 (1.7)	7006 (1.5)	0.01
Co-medications						
Selective serotonin reuptake inhibitors	60006 (13.0)	540642 (8.6)	0.14	60006 (13.0)	55194 (12.0)	0.03
Loperamide	6658 (1.4)	46964 (0.7)	0.07	6658 (1.4)	3597 (0.8)	0.06

IBS refers to irritable bowel syndrome; 95% CI indicates a 95% confidence interval; and HR stands for hazard ratio. In the TriNetX analytics system, data-privacy rules require that any incidence count of 10 or fewer be reported as 10. Furthermore, for the analysis reflected in this table, incident outcome events occurring within the first three months after the index date are not included. The adjusted covariates consist of age, sex (female), race (white), body mass index, comorbidities such as hypertension, hyperlipidemia, diabetes mellitus, anxiety, dissociative and stress-related disorders, somatoform and other nonpsychotic mental disorders, as well as substance use disorders (ICD-10-CM codes F10–F19).

### Primary outcome analysis

**Figure 2:**
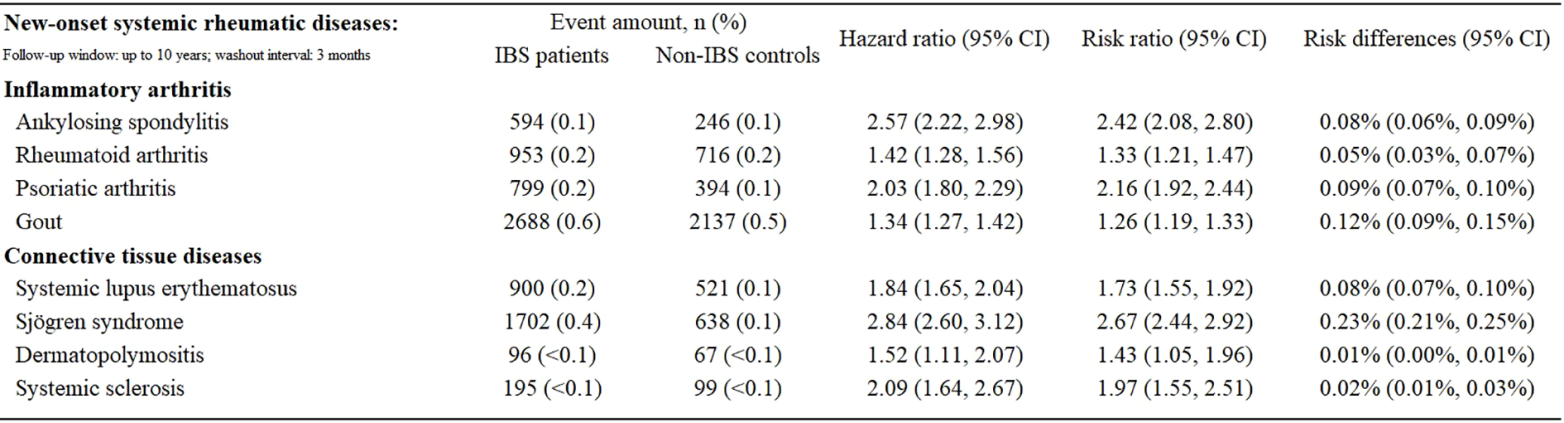
Risk of incident systemic rheumatic diseases among patients with irritable bowel syndrome. Comparison of the risks of newly diagnosed systemic rheumatic diseases between propensity score–matched patients with IBS and individuals without IBS. Outcomes included inflammatory arthritis (ankylosing spondylitis, rheumatoid arthritis, psoriatic arthritis, and gout) and connective tissue diseases (systemic lupus erythematosus, Sjögren syndrome, systemic sclerosis, and dermatomyositis/polymyositis). Effect estimates are presented as hazard ratios with 95% confidence intervals. Risk ratios and risk differences are also shown to provide relative and absolute risk estimates.

During follow-up, IBS was associated with a higher incidence of several systemic rheumatic diseases compared with matched controls (**Figure 2**). Among inflammatory arthritis outcomes, increased risks were observed for ankylosing spondylitis (594 (0.1%) vs. 246 (0.1%); HR 2.57, 95% CI 2.22–2.98), psoriatic arthritis (799 (0.2%) vs. 394 (0.1%); HR 2.03, 95% CI 1.80–2.29), rheumatoid arthritis (953 (0.2%) vs. 716 (0.2%); HR 1.42, 95% CI 1.28–1.56), and gout (2,688 (0.6%) vs. 2,137 (0.5%); HR 1.34, 95% CI 1.27–1.42).

For connective tissue diseases, IBS was associated with higher risks of systemic lupus erythematosus (900 (0.2%) vs. 521 (0.1%); HR 1.84, 95% CI 1.65–2.04), Sjögren syndrome (1,702 (0.4%) vs. 638 (0.1%); HR 2.84, 95% CI 2.60–3.12), systemic sclerosis (195 (<0.1%) vs. 99 (<0.1%); HR 2.09, 95% CI 1.64–2.67), and dermatopolymyositis (96 (<0.1%) vs. 67 (<0.1%); HR 1.52, 95% CI 1.11–2.07). Although relative risks were elevated, absolute event rates were low for several outcomes. Corresponding risk ratios and risk differences are presented in **Figure 2**.

### Sensitivity analyses

The associations were generally consistent across analyses using more restrictive IBS definitions. Similar patterns were observed when IBS was defined by repeat diagnosis within 30 days, more than five IBS diagnostic records, concurrent abdominal pain coding, or concurrent change in bowel habit coding (**Supplementary Tables 3**, **S4**).

Shorter follow-up windows of 1 and 5 years yielded results broadly consistent with the primary 10-year analysis (**Supplementary Table 5**). When the washout interval was extended to 24 and 36 months, the associations also remained directionally similar (**Supplementary Table 6**). These findings suggest that the observed associations were not limited to outcomes occurring shortly after IBS diagnosis, although reverse causation cannot be fully excluded.

### Additional exclusion analyses

After excluding individuals with a history of cholecystectomy, IBS remained associated with higher risks of the evaluated rheumatic disease outcomes. Similar findings were observed after excluding selected abdominal disorders that may mimic IBS, including celiac disease, microscopic colitis, diseases of the esophagus, stomach, and duodenum, and diseases of the peritoneum or retroperitoneum (**Supplementary Table 7**).

### Comparator, utilization, and negative-control analyses

In the utilization-matched model, which additionally incorporated prior ambulatory and inpatient encounter status, the associations remained directionally consistent with the primary analysis (**Supplementary Table 8**). Active comparator analyses using hernia and gastritis or duodenitis as alternative control groups showed attenuated but persistent associations for several outcomes, particularly Sjögren syndrome, ankylosing spondylitis, and psoriatic arthritis. Associations with rheumatoid arthritis, dermatopolymyositis, and systemic sclerosis were weaker in these comparator analyses, suggesting that differential healthcare utilization may have contributed to part of the observed associations (**Supplementary Table 9**). In the negative-control outcome analysis, the estimates were HR 0.93 (95% CI 0.73–1.18) for rickettsioses and HR 0.86 (95% CI 0.73–1.00) for tuberculosis. These analyses did not show a clear positive association between IBS and the selected infectious disease outcomes (**Supplementary Table 10**).

### Cross-dataset validation

Analyses using the TriNetX US Collaborative Network yielded findings broadly consistent with those of the global cohort. Increased risks were observed across inflammatory arthritis and connective tissue disease outcomes, supporting the reproducibility of the overall association pattern in a separate TriNetX sub-dataset (**Supplementary Table 11**).

### Subgroup analyses-age stratification

Age-stratified analyses demonstrated that the association between IBS and subsequent rheumatic diseases was consistently observed across all age groups. Sjögren syndrome showed the strongest association throughout adulthood, with hazard ratios ranging from 3.60 (95% CI 2.35–5.50) among individuals aged 18–29 years to 4.60 (95% CI 3.62–5.85) among those aged 40–49 years, remaining elevated in patients aged 50–64 years (HR 4.24, 95% CI 3.60–5.00) and >65 years (HR 3.79, 95% CI 3.29–4.36). Increased risks of ankylosing spondylitis were also consistently observed across age groups, with the highest estimate in individuals aged 30–39 years (HR 4.78, 95% CI 3.18–7.19). Similarly, systemic lupus erythematosus remained significantly associated with IBS across all age strata, with hazard ratios ranging from 2.27 (95% CI 1.85–2.78) to 3.08 (95% CI 2.53–3.76) (**Tables 2**, **3**).

**Table 2:** Age-stratified associations between irritable bowel syndrome and incident systemic rheumatic diseases in adults aged 18–49 years after propensity score matching.

	18-29 years old			30-39 years old			40-49 years old		
Outcomes	IBS cohort (%)	Control cohort (%)	HR (95% CI)	IBS cohort (%)	Control cohort (%)	HR (95% CI)	IBS cohort (%)	Control cohort (%)	HR (95% CI)
Inflammatory arthritis									
Ankylosing spondylitis	64 (0.1)	21 (<0.1)	3.43 (2.10, 5.62)	131 (0.2)	28 (<0.1)	4.78 (3.18, 7.19)	175 (0.2)	59 (0.1)	3.06 (2.28, 4.11)
Rheumatoid arthritis	26 (<0.1)	17 (<0.1)	1.70 (0.92, 3.13)	96 (0.1)	44 (0.1)	2.23 (1.56, 3.19)	170 (0.2)	80 (0.1)	2.19 (1.68, 2.85)
Psoriatic arthritis	56 (0.1)	26 (<0.1)	2.39 (1.50, 3.80)	140 (0.2)	56 (0.1)	2.57 (1.89, 3.51)	242 (0.3)	88 (0.1)	2.84 (2.23, 3.63)
Gout	32 (<0.1)	29 (<0.1)	1.21 (0.73, 2.01)	184 (0.2)	153 (0.2)	1.23 (0.99, 1.52)	459 (0.6)	259 (0.3)	1.83 (1.57, 2.13)
Connective tissue diseases									
Systemic lupus erythematosus	114 (0.2)	52 (0.1)	2.41 (1.74, 3.35)	239 (0.3)	82 (0.1)	2.99 (2.33, 3.84)	262 (0.3)	90 (0.1)	3.00 (2.36, 3.82)
Sjögren syndrome	91 (0.1)	28 (<0.1)	3.60 (2.35, 5.50)	261 (0.3)	63 (0.1)	4.25 (3.23, 5.60)	365 (0.5)	82 (0.1)	4.60 (3.62, 5.85)
Dermatopolymositis	NA			NA			NA		
Systemic sclerosis	NA			NA			40 (0.1)	12 (<0.1)	3.44 (1.81, 6.56)

IBS refers to irritable bowel syndrome; 95% CI indicates a 95% confidence interval; and HR stands for hazard ratio. In the TriNetX analytics system, data-privacy rules require that any incidence count of 10 or fewer be reported as 10. Furthermore, for the analysis reflected in this table, incident outcome events occurring within the first three months after the index date are not included. The adjusted covariates consist of age, sex (female), race (white), body mass index, comorbidities such as hypertension, hyperlipidemia, diabetes mellitus, anxiety, dissociative and stress-related disorders, somatoform and other nonpsychotic mental disorders, as well as substance use disorders (ICD-10-CM codes F10–F19).

**Table 3:** Age-stratified associations between irritable bowel syndrome and incident systemic rheumatic diseases in adults aged 50-65 years after propensity score matching.

	50-64 years old			Greater than 65 years old		
Outcomes	IBS cohort (%)	Control cohort (%)	HR (95% CI)	IBS cohort (%)	Control cohort (%)	HR (95% CI)
Inflammatory arthritis						
Ankylosing spondylitis	206 (0.2)	75 (0.1)	2.92 (2.24, 3.80)	230 (0.2)	70 (<0.1)	3.40 (2.60, 4.44)
Rheumatoid arthritis	454 (0.4)	239 (0.2)	2.01 (1.72, 2.35)	622 (0.5)	347 (0.3)	1.86 (1.63, 2.12)
Psoriatic arthritis	380 (0.3)	160 (0.1)	2.53 (2.10, 3.04)	284 (0.2)	123 (0.1)	2.39 (1.93, 2.95)
Gout	1043 (1.0)	627 (0.6)	1.78 (1.61, 1.96)	2037 (1.5)	1187 (0.9)	1.78 (1.66, 1.91)
Connective tissue diseases						
Systemic lupus erythematosus	387 (0.4)	133 (0.1)	3.08 (2.53, 3.76)	299 (0.2)	136 (0.1)	2.27 (1.85, 2.78)
Sjögren syndrome	715 (0.7)	179 (0.2)	4.24 (3.60, 5.00)	888 (0.7)	243 (0.2)	3.79 (3.29, 4.36)
Dermatopolymositis	45 (<0.1)	19 (<0.1)	2.50 (1.46, 4.28)	49 (<0.1)	28 (<0.1)	1.81 (1.14, 2.88)
Systemic sclerosis	74 (0.1)	33 (<0.1)	2.37 (1.58, 3.58)	116 (<0.1)	45 (<0.1)	2.66 (1.89, 3.76)

IBS refers to irritable bowel syndrome; 95% CI indicates a 95% confidence interval; and HR stands for hazard ratio. In the TriNetX analytics system, data-privacy rules require that any incidence count of 10 or fewer be reported as 10. Furthermore, for the analysis reflected in this table, incident outcome events occurring within the first three months after the index date are not included. The adjusted covariates consist of age, sex (female), race (white), body mass index, comorbidities such as hypertension, hyperlipidemia, diabetes mellitus, anxiety, dissociative and stress-related disorders, somatoform and other nonpsychotic mental disorders, as well as substance use disorders (ICD-10-CM codes F10–F19).

### Subgroup analyses-sex stratification

When stratified by sex, both men and women with IBS had higher risks of systemic rheumatic diseases than their matched non-IBS counterparts. In male patients, HRs were 3.37 (95% CI 2.56–4.45) for ankylosing spondylitis, 2.01 (95% CI 1.88–2.14) for gout, 3.02 (95% CI 2.00–4.56) for systemic lupus erythematosus, and 6.44 (95% CI 4.51–9.20) for Sjögren syndrome. Among female patients, the corresponding HRs were 4.02 (95% CI 3.39–4.77) for ankylosing spondylitis, 2.06 (95% CI 1.92–2.22) for gout, 3.04 (95% CI 2.73–3.38) for systemic lupus erythematosus, and 4.05 (95% CI 3.70–4.44) for Sjögren syndrome (**Table 4**).

**Table 4:** Sex-stratified associations between irritable bowel syndrome and incident systemic rheumatic diseases after propensity score matching.

	Male			Female		
Outcomes	IBS cohort (%)	Control cohort (%)	HR (95% CI)	IBS cohort (%)	Control cohort (%)	HR (95% CI)
Inflammatory arthritis						
Ankylosing spondylitis	218 (0.1)	65 (<0.1)	3.37 (2.56, 4.45)	646 (0.2)	164 (<0.1)	4.02 (3.39, 4.77)
Rheumatoid arthritis	133 (<0.1)	84 (<0.1)	1.59 (1.21, 2.09)	1292 (0.3)	606 (0.2)	2.18 (1.97, 2.40)
Psoriatic arthritis	219 (0.1)	91 (<0.1)	2.43 (1.91, 3.11)	977 (0.3)	320 (<0.1)	3.12 (2.75, 3.54)
Gout	2608 (1.7)	1307 (0.8)	2.01 (1.88, 2.14)	2229 (0.6)	1104 (0.3)	2.06 (1.92, 2.22)
Connective tissue diseases						
Systemic lupus erythematosus	91 (<0.1)	30 (<0.1)	3.02 (2.00, 4.56)	1360 (0.4)	455 (0.1)	3.04 (2.73, 3.38)
Sjögren syndrome	224 (0.1)	35 (<0.1)	6.44 (4.51, 9.20)	2286 (0.6)	577 (0.2)	4.05 (3.70, 4.44)
Dermatopolymositis	NA			149 (<0.1)	57 (<0.1)	2.66 (1.96, 3.61)
Systemic sclerosis	21 (<0.1)	11 (<0.1)	1.91 (0.92, 3.97)	279 (<0.1)	87 (<0.1)	3.27 (2.57, 4.15)

IBS refers to irritable bowel syndrome; 95% CI indicates a 95% confidence interval; and HR stands for hazard ratio. In the TriNetX analytics system, data-privacy rules require that any incidence count of 10 or fewer be reported as 10. Furthermore, for the analysis reflected in this table, incident outcome events occurring within the first three months after the index date are not included. The adjusted covariates consist of age, sex (female), race (white), body mass index, comorbidities such as hypertension, hyperlipidemia, diabetes mellitus, anxiety, dissociative and stress-related disorders, somatoform and other nonpsychotic mental disorders, as well as substance use disorders (ICD-10-CM codes F10–F19).

### Subgroup analyses-race stratification

Race-stratified analyses further demonstrated that the association between IBS and systemic rheumatic diseases extended across major racial groups, although effect sizes varied. Among White patients, HRs ranged from 1.80 (95% CI 1.69–1.92) for gout to 4.00 (95% CI 3.60–4.44) for Sjögren syndrome. Black patients with IBS had increased risks of rheumatoid arthritis (HR 3.06, 95% CI 2.20–4.25), gout (HR 1.56, 95% CI 1.31–1.86), systemic lupus erythematosus (HR 3.45, 95% CI 2.55–4.65), Sjögren syndrome (HR 3.69, 95% CI 2.63–5.16), and systemic sclerosis (HR 2.08, 95% CI 1.01–4.27). Among Asian patients, elevated risks were particularly significant for gout (HR 1.50, 95% CI 1.12–2.00) and Sjögren syndrome (HR 4.49, 95% CI 2.77–7.28) (**Supplementary Table 12**).

## Discussion

In this large-scale, real-world cohort study derived from a multinational electronic health record network, IBS was associated with a consistently increased risk of incident systemic rheumatic diseases compared with propensity score–matched non-IBS controls. The increased risk was observed across the major categories of inflammatory arthritis and connective tissue diseases, with hazard ratios generally ranging from 1.5 to 3.0. The strongest associations were observed for ankylosing spondylitis and Sjögren syndrome. These associations remained consistent across multiple sensitivity analyses using different washout periods and follow-up durations, supporting the robustness of the findings.

Several pathophysiological mechanisms may contribute to the increased risk of systemic rheumatic diseases observed in patients with IBS. Contemporary models describe IBS as a multifactorial disorder characterized by alterations in gut–brain interactions, visceral hypersensitivity, and, in many patients, evidence of immune activation and low-grade mucosal inflammation (16, 17). Increased mucosal T-cell activation and elevated pro-inflammatory cytokines have been reported in patients with IBS, along with evidence of impaired epithelial barrier function and increased intestinal permeability (18, 19). In parallel, alterations in the gut microbiota are increasingly recognized in IBS, with multiple studies reporting reduced microbial diversity and specific compositional shifts that potentially correlate with symptom severity (20).

Systemic rheumatic diseases are themselves closely linked to perturbations in the gut microbiome and mucosal immunity (21), providing a plausible biological bridge to IBS. A large-scale meta-analysis of observational studies found that gut microbiota dysbiosis is a consistent feature across multiple rheumatic diseases, including RA, spondyloarthritis, and SLE, with shared patterns of reduced microbial diversity and enrichment or depletion of specific taxa (22). In RA, gut microbial dysbiosis and intestinal barrier dysfunction have been proposed to promote Th17-skewed immune responses and autoreactive B-cell activation, thereby contributing to disease onset and progression (22). Likewise, previous studies have reported intestinal dysbiosis in primary Sjögren syndrome, as well as distinct gut and oral microbial profiles in SLE and Sjögren syndrome compared with healthy controls (23, 24). These findings suggest that disturbances in mucosal immunity may play a role in immune-mediated inflammatory pathways. Previous studies have further proposed that alterations in gut microbial composition, epithelial barrier function, and mucosal immune responses may contribute to systemic inflammatory processes. These mechanisms provide plausible biological explanations for the associations observed between IBS and systemic rheumatic diseases in epidemiological studies. However, the present analysis did not include biological measurements capable of directly evaluating these pathways. Consequently, the current findings should be interpreted as evidence of an association rather than confirmation of a specific mechanistic link.

It should be noted that gout differs from the connective tissue diseases and inflammatory arthritis traditionally regarded as autoimmune disorders (25). The pathogenesis of gout is primarily driven by monosodium urate crystal deposition and subsequent activation of innate immune pathways (26). Nevertheless, gout remains one of the most prevalent rheumatic diseases worldwide and is characterized by marked inflammatory responses involving the nucleotide-binding domain, leucine-rich–containing family, pyrin domain–containing-3 (NLRP3) inflammasome and interleukin-1β signaling (26). Therefore, gout was included in the present study as part of the broader spectrum of systemic rheumatic diseases rather than as a classical autoimmune condition.

Although IBS is currently classified as a disorder of gut–brain interaction, we did not observe significant differences in several psychiatric outcomes. This finding should be interpreted cautiously. Psychiatric conditions may be underrecognized in administrative databases, and diagnostic codes may not fully reflect subclinical psychological distress (27). In addition, IBS is a heterogeneous disorder, and the contribution of gut–brain dysregulation varies substantially across individuals. Furthermore, because the control cohort was derived from a healthcare-seeking population rather than healthy community controls, the baseline burden of psychiatric disorders may have been elevated in both groups, potentially attenuating between-group differences (28). Therefore, the absence of significant associations in the current study should not be interpreted as evidence against the gut–brain interaction model of IBS.

From a clinical perspective, the observed associations suggest that rheumatic diseases may merit consideration when individuals with IBS subsequently develop persistent musculoskeletal complaints or other features suggestive of systemic inflammatory disease. Because delayed recognition of rheumatic conditions may adversely affect long-term outcomes, awareness of these potential associations could facilitate timely referral and further evaluation when clinically indicated (29). However, the present findings should not be interpreted as supporting routine screening for rheumatic diseases in all patients with IBS.

Several limitations should be acknowledged. First, disease ascertainment relied on diagnostic codes recorded during routine clinical practice rather than formal adjudication. For IBS, symptom-based criteria and supporting clinical information were unavailable, precluding confirmation of Rome-based diagnoses. Information regarding IBS subtype and clinician specialty was likewise unavailable. Although individuals with inflammatory bowel disease were excluded and additional sensitivity analyses were performed using more restrictive IBS definitions and after excluding selected organic gastrointestinal disorders that may mimic IBS, some degree of diagnostic heterogeneity remains possible. Likewise, laboratory findings and other disease-specific evaluations were unavailable to verify rheumatic disease diagnoses. Although previous studies have reported acceptable performance of coding algorithms for several rheumatic diseases (30, 31), validation within the TriNetX platform remains limited. To reduce potential misclassification, we required at least two IBS-related encounters and performed multiple sensitivity analyses, including a more stringent definition requiring concurrent abdominal symptom coding. Nevertheless, some degree of diagnostic misclassification remains unavoidable and may have influenced the observed associations. Second, as with most real-world studies, residual and unmeasured confounding cannot be excluded despite extensive propensity score matching for demographic characteristics and major comorbidities, particularly with respect to genetic susceptibility and over-the-counter medication use (32–34). Therefore, the findings should be interpreted with caution. Third, information regarding rheumatic disease phenotype and disease activity was limited, precluding evaluation of whether IBS is preferentially associated with specific serological subsets or disease severity trajectories. Fourth, the study population was limited to individuals receiving care within participating healthcare systems. Consequently, patients with limited access to healthcare or milder IBS who did not seek medical attention may have been underrepresented, potentially limiting the generalizability of the findings (28, 35). In addition, differential healthcare utilization may have influenced disease ascertainment. The database did not provide detailed information on longitudinal outpatient visits, specialty consultations, referral patterns, or autoimmune testing practices. Although additional analyses using a healthcare utilization–matched model, a negative-control exposure, and negative-control outcomes produced results generally consistent with the primary analysis, surveillance bias cannot be completely excluded. The attenuation of the associations for rheumatoid arthritis and systemic sclerosis in the hernia comparator analysis further suggests that increased healthcare utilization may have partially contributed to some of the observed associations. Fifth, the possibility of reverse causation should also be considered (36, 37). Certain rheumatic diseases may remain clinically unrecognized for years before diagnosis and may initially present with nonspecific manifestations that overlap with symptoms commonly attributed to IBS. Although analyses using extended washout periods of 24 and 36 months yielded findings similar to those of the primary analysis, these approaches can only partially reduce the possibility of reverse causation. Longer preclinical phases, particularly for Sjögren syndrome and systemic sclerosis, cannot be completely excluded. Sixth, the statistical analyses were constrained by the structure of the TriNetX analytic platform. Although the platform generated hazard ratios, Kaplan–Meier estimates, and log-rank P values, the underlying model specification was not accessible to investigators. Consequently, formal assessment of the proportional hazards assumption was not possible. In addition, death during follow-up was not modeled as a competing event, and competing-risk analyses could not be performed. These limitations may affect the interpretation of time-to-event estimates, particularly for outcomes occurring over prolonged follow-up. Furthermore, because formal power calculations were not available within the TriNetX interface, analyses involving small event counts, particularly subgroup and rare-outcome analyses, should be interpreted with caution. Finally, given the observational nature of the study, causal inferences cannot be drawn (38).

## Conclusion

In conclusion, in this large-scale, multinational real-world study, IBS was associated with an increased risk of a broad range of systemic rheumatic diseases. These findings suggest that individuals with IBS may have a greater risk of subsequent diagnoses of systemic rheumatic diseases and underscore the need for further research to elucidate the biological mechanisms underlying these associations and to determine their clinical implications.

## Data Availability

The datasets presented in this article are not readily available because Data in this study were retrieved from TriNetX Research Network. All data available in the database were administrated by the TriNetX platform. Detailed information can be retrieved at the official website of the research network (https://trinetx.com). Requests to access the datasets should be directed to Official website of the TriNetX research network (https://trinetx.com).
