# Supplementary material for: Risk of systemic rheumatic diseases in people with irritable bowel syndrome: a global-federated cohort analysis
Source: Front Immunol. 2026 Aug 3;17:1779191. doi: 10.3389/fimmu.2026.1779191 (PMC13478090; doi:10.3389/fimmu.2026.1779191)
Supplement: Supplementary file 1 [file SupplementaryFile1.docx]

**Supplementary Tables**

**Table S1. Utilized proxy codes ^a^**

| **Description** | **ICD-10-CM codes** |
| --- | --- |
| *Study population* |  |
| Irritable bowel syndrome | ICD-10-CM: K58 |
| *Outcome: Systemic Rheumatic Diseases* |  |
| Inflammatory arthritis |  |
| Ankylosing spondylitis | ICD-10-CM: M45 |
| Rheumatoid arthritis | ICD-10-CM: M05 |
| Psoriatic arthritis | ICD-10-CM: L40.5 |
| Gout | ICD-10-CM: M10 |
| Connective tissue disease |  |
| Systemic lupus erythematosus | ICD-10-CM: M32 |
| Sjögren syndrome | ICD-10-CM: M35.0 |
| Dermatopolymyositis | ICD-10-CM: M33 |
| Systemic sclerosis | ICD-10-CM: M34 |
| *Other diseases or covariates* |  |
| Encounter for general adult examination | ICD-10-CM: Z00.0 |
| Essential hypertension | ICD-10-CM: I10 |
| Hyperlipidemia | ICD-10-CM: E78.5 |
| Diabetes mellitus | ICD-10-CM: E08-E13 |
| Mental and behavioral disorders due to psychoactive substance use | ICD-10-CM: F10-F19 |
| Persons with potential health hazards related to socioeconomic and psychosocial circumstances | ICD-10-CM: Z55-Z65 |
| Anxiety, dissociative, stress-related, somatoform and other nonpsychotic mental disorders | ICD-10-CM: F40-F48 |
| Major depressive disorder, recurrent | ICD-10-CM: F33 |
| Vitamin D deficiency | ICD-10-CM: E55 |
| Sleep apnea | ICD-10-CM: G47.3 |
| Chronic ischemic heart disease | ICD-10-CM: I25 |
| Chronic kidney disease | ICD-10-CM: N18 |
| *Co-medications* | **RxNorm/ATC codes** |
| Loperamide | RxNorm: 6468 |
| Selective serotonin reuptake inhibitors | ATC code: N06AB |

^a^ICD-10-CM: International Classification of Diseases, Tenth Revision, Clinical Modification;

**Table S2. Description of all applied sensitivity analysis models**

| **Models** | **Description** | **Corresponding table** |
| --- | --- | --- |
| **Applying different definitions of irritable bowel syndrome (IBS)** | | |
| Definition 1 | Only patients being diagnosed of IBS again within 30 days after the first instance of being diagnosed of IBS were included as the IBS group. | **Table S3** |
| Definition 2 | Only patients having records of greater than 5 times of IBS diagnosis were included as the IBS group. |  |
| Definition 3 | Only patients having records of IBS diagnosis and having the diagnosis of abdominal pain (ICD-10-CM: R10.9) were included as the IBS group. | **Table S4** |
| Definition 4 | Only patients having records of IBS diagnosis and having the diagnosis of change in bowel habit (ICD-10-CM: R19.4) were included as the IBS group. |  |
| **Applying different follow-up window after index date** | | |
| 1 year /5 years after index date | Only events linked to study outcomes that occur during the designated follow-up window will be considered. In addition, every sub-analysis applies a uniform three-month washout interval before outcomes are assessed. | **Table S5** |
| **Applying different wash-out interval after index date** | | |
| 24 months/36 months after index date | Outcome events arising within the washout interval are excluded from all further evaluations. Beyond this, each sub-analysis was carried out using a fixed follow-up window of ten years. | **Table S6** |
| **Applying different matching covariates** | | |
| Crude model | Analysis did not undergo propensity score matching. | **Table S7** |
| Matching model 1 | Matching covariates include age at index and sex |  |
| Matching model 2 | Matching covariates included those used in the main analysis: age, sex (female), race (White), body mass index, and comorbidities such as hypertension, hyperlipidemia, diabetes mellitus, anxiety, dissociative and stress-related disorders, somatoform and other nonpsychotic mental disorders, and substance use disorders (ICD-10-CM codes F10–F19), as well as measures of healthcare utilization (inpatient and ambulatory visits). |  |
| Adding additional exclusion criteria |  |  |
| Exclusion of cholecystectomy history | This sensitivity analysis excluded individuals with a history of cholecystectomy and other abdominal dysfunctions to evaluate the robustness of the primary findings. | **Table S8** |
| Exclusion of other abdominal dysfunctions | To minimize potential confounding from pre-existing gastrointestinal conditions that may share symptoms or pathophysiological mechanisms with irritable bowel syndrome (IBS), individuals with a history of other abdominal dysfunctions were excluded from both the IBS and control groups. Other abdominal dysfunctions included celiac disease (K90.0), microscopic colitis (K52.83), diseases of the esophagus, stomach, and duodenum (K20–K31), and diseases of the peritoneum and retroperitoneum (K65–K68). Participants with any record of these diagnoses at any time were excluded from further analysis. |  |
| **Applying active comparators** | | |
| Setting people with hernia/gastritis and duodenitis as control group | This analysis employed patients with hernia/gastritis and duodenitis as an active comparator assess the potential presence of medical surveillance bias and validate the findings. | **Table S9** |
| **Applying negative control outcomes** |  |  |
| Setting outcome as tuberculosis or rickettsioses | To assess the potential presence of residual confounding and systematic bias, a negative-control outcome analysis was conducted using infectious diseases that are not plausibly associated with IBS. The negative-control outcomes included tuberculosis (ICD-10-CM codes A15–A19) and rickettsioses (ICD-10-CM code A79). | **Table S10** |
| **Applying different subset for cross sub-dataset validation** | | |
| US collaborative network | This analysis utilized the US Collaborative Network for cross–sub-dataset validation to ensure that the analytical approach remained consistent across the various component datasets | **Table S11** |

**Table S3. Sensitivity analysis of applying different definitions of irritable bowel syndrome**

|  | Definition 1 | | | Definition 2 | | |
| --- | --- | --- | --- | --- | --- | --- |
| **Outcomes** | IBS cohort (%) | Control cohort (%) | HR (95% CI) | IBS cohort (%) | Control cohort (%) | HR (95% CI) |
| **Inflammatory arthritis** |  |  |  |  |  |  |
| Ankylosing spondylitis | 891 (0.2) | 256 (<0.1) | 3.55 (3.09, 4.07) | 176 (0.3) | 16 (<0.1) | 8.93 (5.35, 14.90) |
| Rheumatoid arthritis | 1525 (0.3) | 736 (0.1) | 2.11 (1.94, 2.31) | 332 (0.6) | 86 (0.2) | 3.13 (2.47, 3.97) |
| Psoriatic arthritis | 1214 (0.2) | 397 (<0.1) | 3.12 (2.79, 3.50) | 221 (0.4) | 44 (<0.1) | 4.06 (2.93, 5.60) |
| Gout | 5034 (0.9) | 2532 (0.5) | 2.03 (1.93, 2.12) | 1049 (1.9) | 262 (0.5) | 3.28 (2.87, 3.76) |
| **Connective tissue diseases** |  |  |  |  |  |  |
| Systemic lupus erythematosus | 1520 (0.3) | 485 (<0.1) | 3.18 (2.88, 3.53) | 309 (0.6) | 56 (0.1) | 4.51 (3.40, 6.00) |
| Sjögren syndrome | 2622 (0.5) | 625 (0.1) | 4.28 (3.92, 4.67) | 591 (1.1) | 69 (0.1) | 6.95 (5.42, 8.92) |
| Dermatopolymositis | 202 (<0.1) | 52 (<0.1) | 3.94 (2.91, 5.34) | NA | | |
| Systemic sclerosis | 313 (<0.1) | 107 (<0.1) | 2.98 (2.39, 3.71) | NA | | |

IBS refers to irritable bowel syndrome; 95% CI indicates a 95% confidence interval; and HR stands for hazard ratio. In the TriNetX analytics system, data-privacy rules require that any incidence count of 10 or fewer be reported as 10. Furthermore, for the analysis reflected in this table, incident outcome events occurring within the first three months after the index date are not included. The adjusted covariates consist of age, sex (female), race (white), body mass index, comorbidities such as hypertension, hyperlipidemia, diabetes mellitus, anxiety, dissociative and stress-related disorders, somatoform and other nonpsychotic mental disorders, as well as substance use disorders (ICD-10-CM codes F10–F19).

**Table S4. Sensitivity analysis of applying different definitions of irritable bowel syndrome-abdominal pain and change of bowel habit**

|  | Definition 3 | | | Definition 4 | | |
| --- | --- | --- | --- | --- | --- | --- |
| **Outcomes** | IBS cohort (%) | Control cohort (%) | HR (95% CI) | IBS cohort (%) | Control cohort (%) | HR (95% CI) |
| **Inflammatory arthritis** |  |  |  |  |  |  |
| Ankylosing spondylitis | 268 (0.2) | 52 (<0.1) | 5.60 (4.16, 7.54) | 44 (0.2) | 13 (0.1) | 3.86 (2.07, 7.18) |
| Rheumatoid arthritis | 437 (0.4) | 162 (0.2) | 2.92 (2.44, 3.50) | 73 (0.4) | 27 (0.2) | 3.06 (1.97, 4.78) |
| Psoriatic arthritis | 332 (0.3) | 104 (0.1) | 3.46 (2.77, 4.31) | 59 (0.3) | 19 (0.1) | 3.59 (2.13, 6.05) |
| Gout | 1036 (1.0) | 487 (0.5) | 2.33 (2.09, 2.60) | 150 (0.9) | 103 (0.6) | 1.67 (1.30, 2.15) |
| **Connective tissue diseases** |  |  |  |  |  |  |
| Systemic lupus erythematosus | 545 (0.5) | 127 (0.1) | 4.63 (3.81, 5.61) | 61 (0.3) | 20 (0.1) | 3.45 (2.08, 5.74) |
| Sjögren syndrome | 779 (0.7) | 121 (0.1) | 7.01 (5.79, 8.49) | 119 (0.7) | 15 (0.1) | 8.79 (5.13, 15.06) |
| Dermatopolymositis |  |  |  | NA | | |
| Systemic sclerosis | 81 (0.1) | 21 (<0.1) | 4.16 (2.57, 6.72) | NA | | |

IBS refers to irritable bowel syndrome; 95% CI indicates a 95% confidence interval; and HR stands for hazard ratio. In the TriNetX analytics system, data-privacy rules require that any incidence count of 10 or fewer be reported as 10. Furthermore, for the analysis reflected in this table, incident outcome events occurring within the first three months after the index date are not included. The adjusted covariates consist of age, sex (female), race (white), body mass index, comorbidities such as hypertension, hyperlipidemia, diabetes mellitus, anxiety, dissociative and stress-related disorders, somatoform and other nonpsychotic mental disorders, as well as substance use disorders (ICD-10-CM codes F10–F19).

**Table S5. Sensitivity analysis of applying different follow-up window after index date**

|  | 1 year | | | 5 years | | |
| --- | --- | --- | --- | --- | --- | --- |
| **Outcomes** | IBS cohort (%) | Control cohort (%) | HR (95% CI) | IBS cohort (%) | Control cohort (%) | HR (95% CI) |
| **Inflammatory arthritis** |  |  |  |  |  |  |
| Ankylosing spondylitis | 59 (<0.1) | 27 (<0.1) | 2.15 (1.36, 3.39) | 346 (0.1) | 124 (<0.1) | 2.78 (2.26, 3.41) |
| Rheumatoid arthritis | 96 (<0.1) | 60 (<0.1) | 1.57 (1.14, 2.17) | 546 (0.1) | 318 (0.1) | 1.71 (1.49, 1.96) |
| Psoriatic arthritis | 72 (<0.1) | 32 (<0.1) | 2.22 (1.46, 3.36) | 434 (0.1) | 187 (<0.1) | 2.31 (1.95, 2.75) |
| Gout | 321 (0.1) | 206 (<0.1) | 1.53 (1.29, 1.83) | 2279 (0.4) | 1230 (0.2) | 1.85 (1.72, 1.98) |
| **Connective tissue diseases** |  |  |  |  |  |  |
| Systemic lupus erythematosus | 104 (<0.1) | 52 (<0.1) | 1.97 (1.41, 2.75) | 580 (0.1) | 256 (<0.1) | 2.26 (1.95, 2.62) |
| Sjögren syndrome | 161 (<0.1) | 61 (<0.1) | 2.60 (1.94, 3.49) | 959 (0.2) | 290 (<0.1) | 3.30 (2.89, 3.76) |
| Dermatopolymositis | NA | | | 75 (<0.1) | 26 (<0.1) | 2.87 (1.84, 4.49) |
| Systemic sclerosis | 29 (<0.1) | 14 (<0.1) | 2.04 (1.08, 3.86) | 128 (<0.1) | 55 (<0.1) | 2.32 (1.69, 3.18) |

IBS refers to irritable bowel syndrome; 95% CI indicates a 95% confidence interval; and HR stands for hazard ratio. In the TriNetX analytics system, data-privacy rules require that any incidence count of 10 or fewer be reported as 10. Furthermore, for the analysis reflected in this table, incident outcome events occurring within the first three months after the index date are not included. The adjusted covariates consist of age, sex (female), race (white), body mass index, comorbidities such as hypertension, hyperlipidemia, diabetes mellitus, anxiety, dissociative and stress-related disorders, somatoform and other nonpsychotic mental disorders, as well as substance use disorders (ICD-10-CM codes F10–F19).

**Table S6. Sensitivity analysis of applying different wash-out intervals after index date**

|  | 2 year | | | 3 years | | |
| --- | --- | --- | --- | --- | --- | --- |
| **Outcomes** | IBS cohort (%) | Control cohort (%) | HR (95% CI) | IBS cohort (%) | Control cohort (%) | HR (95% CI) |
| **Inflammatory arthritis** |  |  |  |  |  |  |
| Ankylosing spondylitis | 543 (0.1) | 226 (<0.1) | 2.47 (2.11, 2.88) | 473 (0.1) | 201 (<0.1) | 2.43 (2.06, 2.87) |
| Rheumatoid arthritis | 892 (0.2) | 605 (0.1) | 1.51 (1.37, 1.68) | 811 (0.2) | 542 (0.1) | 1.54 (1.39, 1.72) |
| Psoriatic arthritis | 788 (0.1) | 386 (<0.1) | 2.10 (1.86, 2.37) | 736 (0.1) | 346 (<0.1) | 2.20 (1.93, 2.50) |
| Gout | 3004 (0.6) | 1894 (0.4) | 1.63 (1.53, 1.72) | 2536 (0.5) | 1625 (0.3) | 1.61 (1.51, 1.71) |
| **Connective tissue diseases** |  |  |  |  |  |  |
| Systemic lupus erythematosus | 862 (0.2) | 411 (<0.1) | 2.15 (1.91, 2.42) | 746 (0.1) | 369 (<0.1) | 2.08 (1.84, 2.36) |
| Sjögren syndrome | 1570 (0.3) | 502 (<0.1) | 3.22 (2.91, 3.56) | 1399 (0.3) | 458 (<0.1) | 3.16 (2.84, 3.51) |
| Dermatopolymositis | 106 (<0.1) | 52 (<0.1) | 2.09 (1.50, 2.91) | 91 (<0.1) | 43 (<0.1) | 2.19 (1.52, 3.14) |
| Systemic sclerosis | 181 (<0.1) | 78 (<0.1) | 2.38 (1.82, 3.10) | 164 (<0.1) | 68 (<0.1) | 2.48 (1.87, 3.30) |

IBS refers to irritable bowel syndrome; 95% CI indicates a 95% confidence interval; and HR stands for hazard ratio. In the TriNetX analytics system, data-privacy rules require that any incidence count of 10 or fewer be reported as 10. Furthermore, for the analysis reflected in this table, incident outcome events occurring within the first three months after the index date are not included. The adjusted covariates consist of age, sex (female), race (white), body mass index, comorbidities such as hypertension, hyperlipidemia, diabetes mellitus, anxiety, dissociative and stress-related disorders, somatoform and other nonpsychotic mental disorders, as well as substance use disorders (ICD-10-CM codes F10–F19).

**Table S7. Sensitivity analysis of adding additional exclusion criteria which could influence rheumatic disorder risk**

|  | Exclusion of cholecystectomy history | | | Exclusion of other abdominal dysfunctions ^a^ | | |
| --- | --- | --- | --- | --- | --- | --- |
| **Outcomes** | IBS cohort (%) | Control cohort (%) | HR (95% CI) | IBS cohort (%) | Control cohort (%) | HR (95% CI) |
| **Inflammatory arthritis** |  |  |  |  |  |  |
| Ankylosing spondylitis | 805 (0.2) | 265 (0.1) | 3.21 (2.79, 3.69) | 241 (0.1) | 93 (<0.1) | 2.90 (2.28, 3.68) |
| Rheumatoid arthritis | 1411 (0.3) | 698 (0.1) | 2.14 (1.95, 2.34) | 382 (0.2) | 271 (0.1) | 1.57 (1.34, 1.83) |
| Psoriatic arthritis | 1116 (0.2) | 442 (0.1) | 2.68 (2.40, 2.99) | 324 (0.1) | 158 (0.1) | 2.29 (1.89, 2.77) |
| Gout | 3908 (0.8) | 2304 (0.5) | 1.80 (1.71, 1.89) | 1048 (0.4) | 825 (0.3) | 1.42 (1.30, 1.56) |
| **Connective tissue diseases** |  |  |  |  |  |  |
| Systemic lupus erythematosus | 1357 (1.6) | 501 (0.6) | 2.86 (2.58, 3.17) | 405 (0.2) | 197 (0.1) | 2.29 (1.93, 2.71) |
| Sjögren syndrome | 2365 (0.5) | 576 (0.1) | 4.35 (3.97, 4.76) | 618 (0.3) | 199 (0.1) | 3.47 (2.96, 4.08) |
| Dermatopolymositis | 130 (<0.1) | 56 (<0.1) | 2.45 (1.79, 3.36) | 38 (<0.1) | 18 (<0.1) | 2.36 (1.34, 4.13) |
| Systemic sclerosis | 278 (0.1) | 107 (<0.1) | 2.74 (2.19, 3.43) | 69 (<0.1) | 27 (<0.1) | 2.85 (1.83, 4.45) |

IBS refers to irritable bowel syndrome; 95% CI indicates a 95% confidence interval; and HR stands for hazard ratio. In the TriNetX analytics system, data-privacy rules require that any incidence count of 10 or fewer be reported as 10. Furthermore, for the analysis reflected in this table, incident outcome events occurring within the first three months after the index date are not included. The wash-out interval were 3 months and the follow-up window were 10 years in this section.

^a^ other abdominal dysfunctions refers to: Celiac disease (K90.0), Microscopic colitis (K52.83), Diseases of esophagus, stomach and duodenum (K20-K31), Diseases of peritoneum and retroperitoneum (K65-K68). In both IBS and control group, individuals having these diagnostic records of all time will be excluded from further analysis.

**Table S8. Sensitivity analysis of applying different matching covariates**

|  | Crude model | | | Model 1 | | | Model 2 | | |
| --- | --- | --- | --- | --- | --- | --- | --- | --- | --- |
| **Outcomes** | IBS cohort (%) | Control cohort (%) | HR (95% CI) | IBS cohort (%) | Control cohort (%) | HR (95% CI) | IBS cohort (%) | Control cohort (%) | HR (95% CI) |
| **Inflammatory arthritis** |  |  |  |  |  |  |  |  |  |
| Ankylosing spondylitis | 665 (0.1) | 2779 (<0.1) | 2.75 (2.53, 2.99) | 631 (0.1) | 233 (<0.1) | 2.70 (2.32, 3.14) | 594 (0.1) | 249 (0.1) | 2.52 (2.18, 2.93) |
| Rheumatoid arthritis | 1020 (0.2) | 7113 (0.1) | 1.65 (1.54, 1.76) | 1000 (0.2) | 682 (0.1) | 1.46 (1.33, 1.61) | 953 (0.2) | 743 (0.2) | 1.35 (1.23, 1.49) |
| Psoriatic arthritis | 876 (0.2) | 4545 (<0.1) | 2.21 (2.06, 2.38) | 861 (0.2) | 394 (<0.1) | 2.18 (1.94, 2.46) | 799 (0.2) | 401 (0.1) | 2.11 (1.87, 2.38) |
| Gout | 3833 (0.7) | 35280 (0.5) | 1.25 (1.21, 1.29) | 3603 (0.7) | 2355 (0.4) | 1.52 (1.45, 1.60) | 2688 (0.6) | 2187 (0.5) | 1.30 (1.23, 1.38) |
| **Connective tissue diseases** |  |  |  |  |  |  |  |  |  |
| Systemic lupus erythematosus | 1013 (0.2) | 4658 (<0.1) | 2.50 (2.33, 2.67) | 988 (0.2) | 471 (<0.1) | 2.09 (1.87, 2.33) | 900 (0.2) | 512 (0.1) | 1.85 (1.66, 2.07) |
| Sjögren syndrome | 1894 (0.3) | 5633 (<0.1) | 3.87 (3.67, 4.07) | 1794 (0.3) | 566 (<0.1) | 3.16 (2.88, 3.48) | 1702 (0.4) | 612 (0.1) | 2.95 (2.69, 3.23) |
| Dermatopolymositis | 134 (<0.1) | 636 (<0.1) | 2.42 (2.01, 2.91) | 131 (<0.1) | 53 (<0.1) | 2.46 (1.79, 3.39) | 96 (<0.1) | 62 (<0.1) | 1.63 (1.18, 2.24) |
| Systemic sclerosis | 222 (<0.1) | 983 (<0.1) | 2.59 (2.24, 3.00) | 217 (<0.1) | 97 (<0.1) | 2.23 (1.75, 2.83) | 195 (<0.1) | 89 (<0.1) | 2.31 (1.80, 2.97) |

IBS refers to irritable bowel syndrome; 95% CI indicates a 95% confidence interval; and HR stands for hazard ratio. In the TriNetX analytics system, data-privacy rules require that any incidence count of 10 or fewer be reported as 10. Furthermore, for the analysis reflected in this table, incident outcome events occurring within the first three months after the index date are not included. The adjusted covariates consist of age, sex (female), race (white), body mass index, comorbidities such as hypertension, hyperlipidemia, diabetes mellitus, anxiety, dissociative and stress-related disorders, somatoform and other nonpsychotic mental disorders, as well as substance use disorders (ICD-10-CM codes F10–F19).

**Table S9. Sensitivity analysis of applying active comparators**

|  | Control group setting as hernia patients | | | Control group setting as gastritis and duodenitis patients | | |
| --- | --- | --- | --- | --- | --- | --- |
| **Outcomes** | IBS cohort (%) | Control cohort (%) | HR (95% CI) | IBS cohort (%) | Control cohort (%) | HR (95% CI) |
| **Inflammatory arthritis** |  |  |  |  |  |  |
| Ankylosing spondylitis | 562 (0.1) | 362 (<0.1) | 1.54 (1.35, 1.76) | 743 (0.2) | 444 (0.1) | 1.52 (1.35, 1.71) |
| Rheumatoid arthritis | 952 (0.2) | 882 (0.2) | 1.07 (0.98, 1.18) | 1329 (0.3) | 970 (0.2) | 1.25 (1.15, 1.36) |
| Psoriatic arthritis | 784 (0.2) | 551 (0.1) | 1.42 (1.27, 1.58) | 1053 (0.2) | 611 (0.1) | 1.57 (1.42, 1.73) |
| Gout | 3555 (0.7) | 2811 (0.6) | 1.26 (1.19, 1.32) | 3812 (0.8) | 2863 (0.6) | 1.22 (1.16, 1.28) |
| **Connective tissue diseases** |  |  |  |  |  |  |
| Systemic lupus erythematosus | 859 (0.2) | 710 (0.1) | 1.20 (1.09, 1.33) | 1270 (0.3) | 846 (0.2) | 1.38 (1.26, 1.50) |
| Sjögren syndrome | 1633 (0.3) | 804 (0.2) | 2.02 (1.86, 2.20) | 2196 (0.5) | 1043 (0.2) | 1.92 (1.79, 2.07) |
| Dermatopolymositis | 119 (<0.1) | 80 (<0.1) | 1.48 (1.11, 1.96) | 118 (<0.1) | 97 (<0.1) | 1.12 (0.85, 1.46) |
| Systemic sclerosis | 192 (<0.1) | 166 (<0.1) | 1.15 (0.93, 1.41) | 249 (0.1) | 184 (<0.1) | 1.24 (1.02, 1.50) |

IBS refers to irritable bowel syndrome; 95% CI indicates a 95% confidence interval; and HR stands for hazard ratio. In the TriNetX analytics system, data-privacy rules require that any incidence count of 10 or fewer be reported as 10. Furthermore, for the analysis reflected in this table, incident outcome events occurring within the first three months after the index date are not included. The adjusted covariates consist of age, sex (female), race (white), body mass index, comorbidities such as hypertension, hyperlipidemia, diabetes mellitus, anxiety, dissociative and stress-related disorders, somatoform and other nonpsychotic mental disorders, as well as substance use disorders (ICD-10-CM codes F10–F19).

**Table S10. Sensitivity analysis of applying negative controls**

|  | Negative control analysis | | |
| --- | --- | --- | --- |
| **Outcomes** | IBS cohort (%) | Control cohort (%) | HR (95% CI) |
| Rickettsioses | 126 (<0.1) | 144 (<0.1) | 0.93 (0.73, 1.18) |
| Tuberculosis | 280 (0.1) | 345 (0.1) | 0.86 (0.73, 1.00) |

IBS refers to irritable bowel syndrome; 95% CI indicates a 95% confidence interval; and HR stands for hazard ratio. In the TriNetX analytics system, data-privacy rules require that any incidence count of 10 or fewer be reported as 10. Furthermore, for the analysis reflected in this table, incident outcome events occurring within the first three months after the index date are not included. The adjusted covariates consist of age, sex (female), race (white), body mass index, comorbidities such as hypertension, hyperlipidemia, diabetes mellitus, anxiety, dissociative and stress-related disorders, somatoform and other nonpsychotic mental disorders, as well as substance use disorders (ICD-10-CM codes F10–F19).

**Table S11. Sensitivity analysis of applying different subset for cross sub-dataset validation**

|  | US collaborative network | | |
| --- | --- | --- | --- |
| **Outcomes** | IBS cohort (%) | Control cohort (%) | HR (95% CI) |
| **Inflammatory arthritis** |  |  |  |
| Ankylosing spondylitis | 649 (0.2) | 221 (<0.1) | 3.06 (2.63, 3.57) |
| Rheumatoid arthritis | 1276 (0.3) | 568 (0.1) | 2.34 (2.12, 2.59) |
| Psoriatic arthritis | 1088 (0.3) | 350 (<0.1) | 3.24 (2.87, 3.66) |
| Gout | 2950 (0.7) | 1743 (0.4) | 1.77 (1.67, 1.88) |
| **Connective tissue diseases** |  |  |  |
| Systemic lupus erythematosus | 1263 (0.3) | 378 (<0.1) | 3.48 (3.10, 3.90) |
| Sjögren syndrome | 2033 (0.5) | 512 (0.1) | 4.15 (3.76, 4.57) |
| Dermatopolymositis | 122 (<0.1) | 58 (<0.1) | 2.19 (1.60, 2.99) |
| Systemic sclerosis | 270 (<0.1) | 92 (<0.1) | 3.05 (2.41, 3.87) |

IBS refers to irritable bowel syndrome; 95% CI indicates a 95% confidence interval; and HR stands for hazard ratio. In the TriNetX analytics system, data-privacy rules require that any incidence count of 10 or fewer be reported as 10. Furthermore, for the analysis reflected in this table, incident outcome events occurring within the first three months after the index date are not included. The adjusted covariates consist of age, sex (female), race (white), body mass index, comorbidities such as hypertension, hyperlipidemia, diabetes mellitus, anxiety, dissociative and stress-related disorders, somatoform and other nonpsychotic mental disorders, as well as substance use disorders (ICD-10-CM codes F10–F19).

**Table S12. Race stratification in risk of new-onset** **systemic rheumatic diseases in IBS patients**

|  | White | | | Black | | | Asian | | |
| --- | --- | --- | --- | --- | --- | --- | --- | --- | --- |
| **Outcomes** | IBS cohort (%) | Control cohort (%) | HR (95% CI) | IBS cohort (%) | Control cohort (%) | HR (95% CI) | IBS cohort (%) | Control cohort (%) | HR (95% CI) |
| **Inflammatory arthritis** |  |  |  |  |  |  |  |  |  |
| Ankylosing spondylitis | 594 (0.2) | 159 (<0.1) | 3.91 (3.28, 4.66) | 31 (0.1) | 12 (<0.1) | 2.57 (1.32, 5.01) | NA | | |
| Rheumatoid arthritis | 1030 (0.3) | 507 (0.1) | 2.13 (1.91, 2.37) | 144 (0.4) | 47 (0.1) | 3.06 (2.20, 4.25) | 19 (0.2) | 14 (0.1) | 1.31 (0.66, 2.61) |
| Psoriatic arthritis | 978 (0.3) | 381 (0.1) | 2.69 (2.39, 3.03) | NA | | |  |  | NA |
| Gout | 2433 (0.7) | 1419 (0.4) | 1.80 (1.69, 1.92) | 327 (1.0) | 209 (0.6) | 1.56 (1.31, 1.86) | 118 (0.9) | 75 (0.6) | 1.50 (1.12, 2.00) |
| **Connective tissue diseases** |  |  |  |  |  |  |  |  |  |
| Systemic lupus erythematosus | 948 (0.3) | 322 (<0.1) | 3.08 (2.71, 3.49) | 190 (0.6) | 55 (0.2) | 3.45 (2.55, 4.65) | NA | | |
| Sjögren syndrome | 1691 (0.5) | 443 (0.1) | 4.00 (3.60, 4.44) | 159 (0.5) | 43 (0.1) | 3.69 (2.63, 5.16) | 95 (0.8) | 20 (0.2) | 4.49 (2.77, 7.28) |
| Dermatopolymositis | 105 (<0.1) | 37 (<0.1) | 2.97 (2.04, 4.31) | NA | | | NA | | |
| Systemic sclerosis | 224 (<0.1) | 76 (<0.1) | 3.08 (2.37, 3.99) | 23 (<0.1) | 11 (<0.1) | 2.08 (1.01, 4.27) | NA | | |

IBS refers to irritable bowel syndrome; 95% CI indicates a 95% confidence interval; and HR stands for hazard ratio. In the TriNetX analytics system, data-privacy rules require that any incidence count of 10 or fewer be reported as 10. Furthermore, for the analysis reflected in this table, incident outcome events occurring within the first three months after the index date are not included. The adjusted covariates consist of age, sex (female), race (white), body mass index, comorbidities such as hypertension, hyperlipidemia, diabetes mellitus, anxiety, dissociative and stress-related disorders, somatoform and other nonpsychotic mental disorders, as well as substance use disorders (ICD-10-CM codes F10–F19).

**Supplementary statement regarding data access and analysis date:**

The original analyses for the first submitted manuscript were performed via the TriNetX analytic tool (the Advanced analytics function, “compare outcome” analysis) between November 28, 2025 and December 1, 2025. During revision, the primary analysis (as presented in Figure 2) was rerun on June 23, 2026 to incorporate additional absolute measures, including risk ratios and risk differences, in response to the reviewer’s comments. The cohort definitions were not changed for this rerun. Additional revision analyses, including the active comparator analyses, negative-control outcome analyses, utilization-matched model, and additional exclusion analyses, were performed using the same analytic tool between June 2, 2026 and June 7, 2026. These analyses used the same prespecified disease definitions and eligibility criteria described in the Methods section.
